# The relationship between social capital and postpartum depression symptoms of lactating women in minority areas—A cross-sectional study from Guangxi, China

**DOI:** 10.3389/fpsyg.2022.905028

**Published:** 2022-11-15

**Authors:** Yinghua Qin, Pengfei Guo, Jiacheng Li, Jingjing Liu, Shengchao Jiang, Feng Yang, Rizhen Wang, Jiahui Wang, Huan Liu, Xin Zhang, Kexin Wang, Qunhong Wu, Wuxiang Shi

**Affiliations:** ^1^Department of Social Medicine, School of Public Health, Health Management College, Harbin Medical University, Harbin, China; ^2^Department of Health Economy and Social Security, College of Humanities and Management, Guilin Medical University, Guilin, China

**Keywords:** postpartum depression symptoms, social capital, lactating women, minority areas, China

## Abstract

**Background:**

Postpartum depression (PPD) is the most common mental illness affecting women during lactation, and good social capital is considered a protective factor. This study aimed to investigate PPD symptoms, and explore the relationships between social capital and PPD symptoms of lactating women in southwest minority areas in China.

**Materials and methods:**

This cross-sectional study was conducted among 413 lactating women in Guangxi, China. Data were collected using the Edinburgh Postnatal Depression Scale and the Chinese version of the Social Capital Assessment Questionnaire. Hierarchical regression analysis was conducted to explore the factors influencing PPD symptoms, and a structural equation model was used to examine how social participation and cognitive social capital mediated PPD symptoms.

**Results:**

The total prevalence of PPD symptoms (score > 12) was 16.46%, and that of mild depression symptoms (9–12 score) was 22.03%. Nine variables predicted PPD symptoms and explained 71.6% of the variance in the regression model: higher age, lack of medical security, fixed occupation, breastfeeding time, self-caregiver, maternity leave, social participation, social trust, and social reciprocity. Furthermore, cognitive social capital mediated the relationship between social participation and PPD symptoms, with a mediation effect rate was 44.00%.

**Conclusion:**

The findings of this study highlight that social capital, support from family members, maternity leave, and medical insurance play protective roles in the PPD symptoms of lactating women. It is necessary to improve social capital as a key strategy for interventions for PPD symptoms, and active social participation activities are critical to reducing PPD symptoms among lactating women in minority areas.

## Introduction

Lactation is a high-risk period for women to experience postpartum depression (PPD). PPD symptoms usually occur 4–6 weeks after a baby is born and usually manifest as lack of sleep, poor appetite, anxiety, irritability, and sadness, which even increases the risk of suicide in postpartum women (Orsolini et al., 2016; Xiong and Deng, 2020b). The intensity and duration of the PPD symptoms differ among women. If not detected and intervened on time, PPD symptoms could last more than 1 year in lactation and even become a lifelong condition (Tarar et al., 2021). Some studies have shown a correlation between mothers’ depression symptoms, infants’ systemic inflammation, adolescent growth retardation, and adolescent behavioral problems (Deave et al., 2008; Posmontier and Waite, 2011; Mirhosseini et al., 2015; Decaro et al., 2016; Junge et al., 2017). Therefore, PPD symptoms affect the mother’s physical and mental health and have harmful effects on family relationships and the child’s development.

PPD symptoms is widely regarded as a cross-cultural, serious public health problem that needs to be solved urgently (Wisner et al., 2006; Howard et al., 2014). According to the World Health Organization, approximately 13% of women worldwide suffer from PPD after childbirth, and the proportion in developing countries is approximately 19.8% (Fisher et al., 2012; Tang et al., 2016). Recent researches show that in some economically backward developing countries, the prevalence of PPD ranges from 14.7 to 31.8%, depending on the factors such as the postpartum period at the time of screening PPD symptoms, regional cultural characteristics, scales used, and positive standards (Murray et al., 2015; Inthaphatha et al., 2020; Oskovi-Kaplan et al., 2021).

Several studies have explored the risk factors for PPD symptoms (Wu et al., 2014; Cankorur et al., 2015; Murray et al., 2015; Chen et al., 2018; Yörük et al., 2020; Seymour-Smith et al., 2021). These risk factors include age, low education level, and low socioeconomic status in sociodemographic characteristics. Further, low marital satisfaction, poor mother-in-law relationships, and stressful life events in family relationships also contribute to PPD. Additionally, negative childbirth experiences; and loss of social support in the postpartum period may cause or worsen PPD symptoms. Therefore, there is an urgent need to take measures to intervene in PPD. One concept proposed to augment the public health approach to PPD is social capital (SC), a combination of perspectives that focus on community, cohesion, group membership, and connections (Smith and Lincoln, 2011).

According to Coleman and Putnam, SC is an inherent social resource in social structures and connections (Coleman, 1988; Stern and Putnam, 1993). In these structures and connections, individuals have a membership and reach a consensus on social norms and cultural values, including many aspects such as citizen participation, collective action, social cohesion, trust, and available social resources (Siisiäinen, 2003). SC is divided into two important dimensions, namely cognitive social capital (CSC) and structural social capital (SSC), which are conceptual structures related to this research (De Silva et al., 2007; Forsman et al., 2012). CSC is a psychological framework that assumes the accessibility of social resources; it is an individual’s expectation of trust in social members, group cohesion, and social reciprocity, which affect the degree of people’s participation in social activities (Islam et al., 2006; Ehsan and De Silva, 2015). SSC includes observable elements, such as the number and attributes of social network members, activities, and frequency of social participation (Wilmot and Dauner, 2017). Hence, according to the traditional definition and the practice of previous research scholars, CSC can be measured by social trust (ST) in the community and social reciprocity (SR) of neighbors; and SSC can be measured by social participation (SP) and social network (SN). These observational indicators have often been used to reflect the impact of social capital on physical and mental health and depression (Forsman et al., 2012; Kritsotakis et al., 2013; Zhou et al., 2017b).

In the internal structure of social capital, CSC and SSC are two closely related concepts, and there may be a causal relationship with each other. We tend to argue that an individual’s objective behaviors will have an impact on his subjective cognition, and when the external SSC changes, the subjective CSC may change accordingly, thus affecting the individual’s mental health. This view has been confirmed in previous studies (Lu and Peng, 2019; Wong et al., 2019; Sun and Lu, 2020; Yang, 2021). Scholars posited that a vicious cycle between low social participation and low levels of social trust and reciprocity, eventually led to a decline in overall SC and affected dividual mental health. Based on this, Lu explored the mediating role of CSC on the relationship between SSC and the depressive symptoms of the elderly (Lu and Peng, 2019).

Studies have shown a strong correlation between SC and mental health (Hamano et al., 2010; Lamarca et al., 2014; Strange et al., 2016; Quick et al., 2021). Residents who believe their SC is low are more likely to report poor mental health than their counterparts. However, SC also has a protective effect on mothers’ well-being (Mulvaney and Kendrick, 2005; Held and Cuellar, 2016). Research on the relationship between SC and PPD has been conducted in different countries. Theoretical concepts have been proposed, including social support networks, SC, and social cohesion, to explain the spatial clustering of maternal depression (Eastwood et al., 2016). Studies have explored the impact of certain aspects of SC on the mental health of postpartum women, such as social participation, community network relations, and community social capital, and found a negative correlation between high SC and depression during pregnancy and breastfeeding (Mulvaney and Kendrick, 2005; Kritsotakis et al., 2013; Strange et al., 2016; Zhou et al., 2018; Gu, 2020).

Chinese women also face PPD, the incidence in economically developed regions is as high as 23.2–34.0% (Liu et al., 2020; Xiong and Deng, 2020b). As a relational society, Chinese society has always had a traditional postpartum culture. What went wrong regarding PPD symptoms? A few studies have focused on exploring the relationship between PPD symptoms and SC in Chinese women during the perinatal or traditional confinement period “doing the month”; “doing the month” is a custom that the mother is requested to isolate at home for 1–2 months after delivery to nurture mother’s body as well and breastfeed the newborn. In a cross-sectional study, Zhou Chi al. confirmed the impact of SC on prenatal depression (Zhou et al., 2017b,Zhou et al., 2018). China’s fertility culture, in addition to the “doing month” culture, also includes breastfeeding for a longer period. During this period, mothers suspend work and devote more time and energy to feeding their children (Ding et al., 2018; Li et al., 2020; Guo et al., 2021; Wang et al., 2021). However, no study has explored the mechanism of SSC and CSC on PPD symptoms during breastfeeding. This is what this study aims to explore.

Some evidence suggests that the incidence of depression in low-income minority women is high. A survey of immigrant mothers in Australia showed that 35% of mothers in multi-ethnic areas and with low socioeconomic status suffered from severe depression (Eastwood et al., 2013). China is a country with multi-ethnic integration; the diversity of ethnic populations in the southwestern region is relatively prominent, and the region is also an economically backward area compared to the eastern coastal area (Huang et al., 2020). Health inequality among ethnic groups is obvious in maternal and child health care. Although studies have focused on the PPD of women in the eastern, central, and northwestern regions, few studies have been conducted in the southwestern minority regions (Gao et al., 2017).

Based on the literature and social capital theory, we propose two hypotheses: 1. there may have directed effect of four indicators of cognitive and structural social capital (ST, SR, SN, SP) on PPD symptoms, lower social capital would be associated with higher level PPD symptoms; 2. we also further tested the mediating role of cognitive social capital between structural social capital and PPD symptoms. Hence, this study aimed to understand the status of PPD symptoms in lactating women in minority areas in southwestern China and to explore the possible associations between PPD symptoms and SC.

## Materials and methods

### Study setting

This research was conducted in the Guangxi Zhuang Autonomous Region of southwestern China. The geographical features of this area are dominated by mountainous areas bordering many Southeast Asian countries. Guangxi is a typical gathering place for ethnic minorities and has the largest ethnic minority population in China. The proportion of ethnic minorities dominated by Zhuang nationality reached 39.00%(Wu et al., 2020). In Guangxi, most pregnant women give birth at local hospitals and return with babies for regular check-ups during breastfeeding.

### Study design

According to the WHO’s breastfeeding guidelines, infants should be breastfed for the first 6 months of life, and breastfeeding continues for up to 2 years of age or beyond. Therefore, the lactation period for postpartum women in this study ranged from the birth of the newborn to 24 months. Two-stage sampling was adopted to conduct a cross-sectional survey; data were collected from December 2018 to May 2019. Three cities in Guangxi were randomly selected, and a maternity hospital pediatric outpatient clinic was selected from each city as a recruitment point. Random sampling was used to recruit lactating women. Before conducting the questionnaire survey, the content of the research project was introduced to each potential participant. To participate in the study, the women must be: (1) Breastfeeding; (2) registered at a Guangxi household and living there for over a year; (3) 18 years or older; (4) voluntary participants; and (5) must have reading comprehension skills. After the respondents signed the informed consent form, the investigators gave them paper questionnaires to complete, which were collected after completion. A total of 500 questionnaires were distributed; incomplete questionnaires were excluded after quality reviews by three researchers, and 413 valid questionnaires were returned, with an effective response rate of 82.60%. The study protocol was conducted in accordance with the Helsinki Declaration and was approved by the Medical Ethics Committee of Guilin Medical University (reference No: GLMC201806301).

## Measurements

### Measurement of postpartum depression symptoms

The Edinburgh Postnatal Depression Scale (EPDS) is widely used to measure the symptoms of depression in postpartum women. The scale consists of ten self-reported items, each with a score of 0–3. The reliability and validity of the Chinese version of the EPDS (C-EPDS) for Chinese maternal measurement have been verified (Cronbach’s α coefficient, 0.87; validity, 0.79) (Heh, 2001; Chen et al., 2018). To measure PPD symptoms, 9 points and 13 points are critical values; 0–8 points indicate no PPD symptoms, 9–12 points indicate the presence of mild PPD symptoms, and 13–30 points indicate the presence of PPD symptoms, insinuating the possibility of postpartum depression (Zhou et al., 2017b). In this study, the C-EPDS was used to measure PPD symptoms of lactating women; Cronbach’s α of the scale was 0.806.

### Measurement of social capital

The Social Capital scale was revised using the World Bank’s Social Capital Assessment Tool and the social capital scale of perinatal women in eastern China (Grootaert and Bastelaer, 2002; Zhou et al., 2017a). In a previous study, Cronbach’s α for the Chinese version of the Social Capital Scale was 0.838. SC can be divided into cognitive and structural forms. CSC was measured using the two sub-dimensions of social trust (ST) and social reciprocity (SR). ST was assessed using six items to investigate the degree of trust and cohesion among friends, neighbors, and healthcare workers. SR was assessed using six items inquiring about the degree of reciprocity among friends, neighbors, and healthcare workers. A five-point system (1–5 points) was used to evaluate each item of ST and SR, and scores were calculated separately. The higher the total score, the higher the level of ST/SR.

SSC was measured using the two dimensions of social participation (SP) and social network (SN). SP was assessed using nine items to reflect the frequency of participation in different activities and the degree of the activity. A five-point system (1–5 points) was used to evaluate each item of SP and calculate the total score; the higher the total score, the higher the level of social participation. SN was comprehensively reflected by the number of occupation types and the prestige status of the occupations that the respondents encountered in social interaction. This included the diversity of social networks (the number of different occupations of social members), the prestige of the occupation (the highest professional prestige score of a social member), and the prestige range in the social network (the highest prestige score minus the lowest prestige score). Occupational prestige refers to the results of Li Chunling’s hierarchical measurement of contemporary Chinese social prestige, which covers 81 occupations in China. The occupational prestige score ranges from 9.73 to 90.15 (Li, 2005). As the measurement units of the three indicators of SN were inconsistent, standardized scores were used to calculate the total score. In this study, the Cronbach’s α coefficient for the social capital scale was 0.802.

*Covariate* control variables included sociodemographic characteristics (household register, ethnicity, and age), socioeconomic status (education level, monthly family income, medical security, and career status), and factors during breastfeeding (length of maternity leave, length of breastfeeding period, type of primary caregivers, and the number of caregivers). The variable assignments are listed in Table 1.

**TABLE 1:** Basic characteristics of samples and distribution of Edinburgh Postnatal Depression Scale scores.

Variate	*n* (%)	Mean ± SD	*T*/*F*	*P*
Ethnicity				
Minority	139 (33.66)	7.71 (3.97)	0.495	0.621
Han	274 (66.34)	7.92 (4.06)		
Household register				
Rural	234 (56.66)	8.8 (4.24)	–5.685	*P* < 0.001
City	179 (43.34)	6.61 (3.34)		
Education level				
Secondary school and below	74 (17.92)	11.27 (4.49)	46.773	*P* < 0.001
Senior high school	126 (30.51)	8.06 (3.49)		
College degree and above	213 (51.57)	6.54 (3.39)		
Family monthly income				
≤3,500 CNY	82 (19.85)	10.9 (4.05)	37.361	*P* < 0.001
3,500–5,000 CNY	115 (27.85)	9.18 (3.51)		
5,000–6,500 CNY	83 (20.1)	6.36 (3.55)		
6,500–8,000 CNY	78 (18.89)	6.24 (2.98)		
>8,000 CNY	55 (13.32)	5.04 (2.84)		
Type of medical security				
None	30 (7.26)	11.77 (5)	35.446	*P* < 0.001
Basic medical insurance for urban and rural residents	141 (34.14)	9.35 (3.46)		
Basic medical insurance for urban employees	123 (29.78)	7.28 (3.43)		
Additional commercial insurance	119 (28.81)	5.68 (3.51)		
Career status				
Fixed occupation	196 (47.46)	6.93 (3.34)	10.176	*P* < 0.001
Freelance	113 (27.36)	8.78 (4.19)		
No Occupation	104 (25.18%)	8.57 (4.64)		
Primary caregiver				
Oneself	116 (28.09)	9.32 (4.359)	9.728	*P* < 0.001
Husband	101 (24.46)	8.05 (3.991)		
Parents in-law	110 (26.63)	7.07 (3.487)		
Parents	48 (11.62)	7.73 (3.734)		
Nanny	38 (9.2)	5.24 (2.999)		
Maternity leave				
None	212 (51.33)	8.5 (4.41)	14.971	*P* < 0.001
Within 98 days	108 (26.15)	8.25 (3.31)		
98 days or more	93 (22.52)	5.91 (3.21)		

### Analytical strategies

Data analysis was conducted using SPSS 23.0 and Amos24.0. The specific steps were as follows: Descriptive analysis of the basic situation of the research object; *t*-test and analysis of variance were used to analyze the association between different demographic and social characteristics and PPD symptoms; Pearson correlation analysis was adopted to evaluate the correlation between SC and PPD symptoms; and a hierarchical regression analysis model was used to evaluate the effects of sociodemographic characteristics, factors during breastfeeding, and social capital on PPD symptoms in lactating women.

**FIGURE 1:**
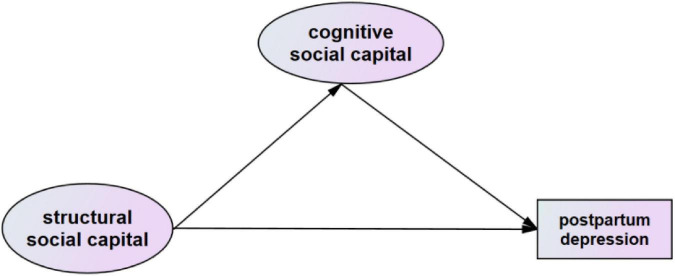
Conceptual model.

Based on the relevant experience in the literature and the results of hierarchical regression analysis, we developed a conceptual model, as shown in Figure 1. The figure posits that social capital’s cognitive dimension CSC mediates between its structural dimension SSC and PPD symptoms. A structural equation model (SEM) was established to evaluate the mediating role of CSC of lactating women between SSC and PPD symptoms. And the observation indicators SP, SN, ST and SR corresponding to these two dimensions were used to verify this model. The following indicators and standards were used to evaluate the fitness of SEM: chi-square value/degrees of freedom (2–5), root mean square error of approximation (RMSEA) (<0.08, “reasonable fit”), comparative fit index (CFI) (>0.9). The bootstrap method was used to test the mediation effect (*n* = 5,000), and statistical significance was set at *P* < 0.05.

## Results

### The characteristics of the samples

The 413 respondents were between the ages of 19 and 43 (28.33 ± 4.10), ethnic minorities accounted for 33.66%, and rural household registration accounted for 56.66%. Nearly half of the respondents had a high school education or below, and the distribution of monthly household income was relatively even, Further, 7.26% of participants were not enrolled in basic medical insurance; The lactation period ranged from 1 to 531 days (173.44 ± 109.46), and 51.33% of lactating women did not have maternity leave. The EPDS scores of respondents ranged from 0 to 27 (7.87 ± 4.02), 254 participants (61.50%) did not have PPD symptoms, and the prevalence of PPD symptoms was 16.46%. The distribution of PPD symptoms among the other characteristic variables was statistically significant (*P* < 0.05) (see Tables 1 and 2 for details).

**TABLE 2:** Distribution of postpartum depression symptoms in different ethnic groups.

Variate	No depression symptoms (<9)	Mild depression symptoms (9–12)	Postpartum depression symptoms (>12)	*χ^2^*	*P*
Minority	93 (66.91%)	27 (19.42%)	19 (13.67%)	2.637	0.267
Han	161 (58.76%)	64 (23.36%)	49 (17.88%)		
Total	254 (61.50%)	91 (22.03%)	68 (16.46%)		

### The results of correlation analysis

Table 3 shows that the age of lactating women and the length of the breastfeeding period were positively correlated with PPD symptoms scores, and the number of caregivers after childbirth was negatively correlated with PPD symptoms. The level of SC was described as ST = 22.26 ± 3.165, SR = 19.23 ± 3.002, SP = 20.16 ± 4.830, and SN = 0.000 ± 2.260, each of them was negatively correlated with PPD symptoms.

**TABLE 3:** Means, standard deviations, and correlation between variates.

Variate	Mean ± SD	Age	Breastfeeding time (day)	Number of caregivers	ST	SR	SP	SW	PPD symptoms
Age	28.33 (4.10)	1							
Breastfeeding time (day)	173.44 (109.46)	0.116*	1						
Number of caregivers	3.02 (1.01)	–0.017	–0.069	1					
ST	22.26 (3.17)	–0.004	−0.262**	0.195**	1				
SR	19.23 (3.00)	−0.121*	−0.126*	0.204**	0.555**	1			
SP	20.16 (4.83)	–0.034	−0.200**	0.273**	0.725**	0.510**	1		
SW	0.00 (2.26)	0.008	–0.050	0.262**	0.459**	0.203**	0.631**	1	
PPD symptoms	7.85 (4.02)	0.230**	0.263**	−0.276**	−0.695**	−0.605**	−0.738**	−0.483**	1

*Correlation is significant at the 0.05 level (2-tailed).

**Correlation is significant at the 0.01 level (2-tailed).

### The results of hierarchical regression analysis of factors affecting postpartum depression symptoms

Table 4 presents the results of the hierarchical regression analysis. In the model, the Durbin-Watson value was 1.977, and the variance inflation factors (VIF) were less than 10, indicating that the residuals were uncorrelated and the explanatory variables had no multicollinearity.

**TABLE 4:** Hierarchical regression analysis of variables affecting postpartum depression symptoms.

Dimension	Variate	Model 1					Model 2					Model 3				
		*B*	β	95% CI for *B*		*P*	*B*	β	95% CI for B		*P*	*B*	β	95% CI for B		*P*
				Lower	Upper				Lower	Upper				Lower	Upper	
Sociodemographic	Age	0.296	0.301	0.220	0.372	0.000	0.261	0.266	0.187	0.336	0.000	0.182	0.186	0.128	0.237	0.000
Characteristics	Household register	–0.450	–0.055	–1.145	0.245	0.204	–0.431	–0.053	–1.114	0.252	0.216	–0.183	–0.023	–0.682	0.315	0.470
	Education level	–0.633	–0.120	–1.252	–0.014	0.045	–0.453	–0.086	–1.052	0.146	0.138	0.097	0.018	–0.343	0.538	0.664
	Family monthly income	–1.200	–0.394	–1.530	–0.870	0.000	–1.077	–0.354	–1.412	–0.741	0.000	–0.103	–0.034	–0.385	0.179	0.473
	Type of medical security (Ref: None)															
	Basic Medical Insurance for Urban and Rural Residents	–1.656	–0.195	–2.930	–0.382	0.011	–1.713	–0.202	–2.944	–0.483	0.006	–1.270	–0.150	–2.164	–0.376	0.006
	Basic Medical Insurance for Urban Employees	–0.802	–0.274	–1.317	–0.288	0.002	–0.765	–0.261	–1.261	–0.269	0.003	–0.621	–0.212	–0.982	–0.261	0.001
	Additional commercial insurance	–3.231	–0.364	–4.787	–1.674	0.000	–2.870	–0.323	–4.396	–1.344	0.000	–1.623	–0.183	–2.739	–0.506	0.005
	Career status (Ref: Fixed occupation)															
	Freelance	–0.816	–0.090	–1.746	0.114	0.085	–1.422	–0.158	–2.563	–0.281	0.015	–1.280	–0.142	–2.112	–0.448	0.003
	No Occupation	–1.692	–0.183	–2.659	–0.725	0.001	–2.090	–0.226	–3.307	–0.873	0.001	–0.661	–0.071	–1.578	0.256	0.157
Factors during	Breastfeeding time (day)						0.006	0.161	0.003	0.009	0.000	0.003	0.079	0.001	0.005	0.006
breastfeeding	Primary caregiver (Ref: Oneself)															
	Husband						–0.987	–0.106	–1.818	–0.155	0.020	–0.438	–0.047	–1.045	0.170	0.157
	Parents in-law						–1.146	–0.126	–1.988	–0.304	0.008	–1.052	–0.116	–1.664	–0.440	0.001
	Parents						–0.175	–0.014	–1.264	0.915	0.753	–0.882	–0.070	–1.678	–0.087	0.030
	Nanny						–1.311	–0.094	–2.517	–0.105	0.033	–1.226	–0.088	–2.103	–0.348	0.006
	Number of caregivers						–0.501	–0.126	–0.813	–0.188	0.002	–0.194	–0.049	–0.425	0.037	0.099
	Maternity leave						–0.494	–0.100	–1.127	0.139	0.126	–0.532	–0.107	–0.994	–0.070	0.024
Social capital	ST											–0.277	–0.217	–0.388	–0.166	0.000
	SR											–0.357	–0.266	–0.452	–0.262	0.000
	SP											–0.243	–0.292	–0.328	–0.158	0.000
	SN											–0.080	–0.052	–0.194	0.034	0.170
	(Constant)	7.341		4.572	10.109	0.000	9.716		6.489	12.943	0.000	24.308		21.343	27.273	0.000
	*F*	29.456					20.636					49.371				
	*R* ^2^	0.397					0.455					0.716				
	Adjusted *R*^2^	0.383					0.433					0.701				
	△*R*^2^	0.397					0.058					0.261				
	Sig. of the model	<0.001					<0.001					<0.001				

B, unstandardized beta; β, the standardized beta.

In Model 1, demographic and sociological factors were included. Age, family monthly income, and medical security of lactating women had statistically significant effects on PPD symptoms (*P* < 0.001); Model 1 significantly explained 39.7% of the variance in PPD symptoms (*F* = 29.456, *P* < 0.001). In Model 2, the individual factors, including breastfeeding time, primary caregiver, number of caregivers, and maternity leave of women during the breastfeeding period were added; Model 2 significantly explained 45.5% of the variance in PPD symptoms (*F* = 20.636, *P* < 0.001).

In Model 3, SC was added, leading to an improvement with a significant change in *R*^2^ of 26.1% (*F* = 49.371, *P* < 0.001). This model explained 71.6% of the variance and revealed nine variables that contributed significantly to PPD symptoms. Among SC, ST (*B* = –0.277, 95%CI = –0.388, -0.166), SR (*B* = –0.357, 95%CI = –0.452, -0.262), and SP (*B* = –0.243, 95%CI = –0.328, -0.158) had statistically significant effects on PPD symptoms, whereas SN had no significant effect.

By comparing the results of Models 3 and 1/2, after adding the four dimensions of SC, the regression coefficient of age changed from 0.296 to 0.182, the regression coefficient of the breastfeeding period decreased from 0.006 to 0.003, and the regression coefficients of all types of medical insurance increased. This meant that the negative effect of no insurance was relatively reduced. Further, the regression coefficients of parents-in-law care, parental care, and nanny care increased, which meant that the negative impact of self-care was relatively reduced. The length of maternity leave had a significant protective effect on PPD symptoms; however, compared with regular professionals, freelancers were significantly negatively correlated with PPD symptoms. Overall, the results showed that ST, SR, and SP played protective roles in regulating PPD symptoms in lactating women.

### Mediating effect of cognitive social capital on social participation and postpartum depression symptoms

Based on the results of the hierarchical regression analysis, we further examine how social capital acts on PPD symptoms. According to the fitting index requirements of SEM, we excluded the two mediation models in which the SSC dimension is composed of the observation indexes SN and SP, and the SSC dimension is only represented by the observation index SN. An SEM was further adopted to test the mediating effect of the CSC on the relationship between SP and PPD symptoms of lactating women, in which SP is the observation index of SSC. The fit indices for the SEM were: *χ^2^/df* = 3.563, RMSEA = 0.079, CFI = 0.981, NIF = 0.981, and AGFI = 0.931, the fit of the model is fine.

**FIGURE 2:**
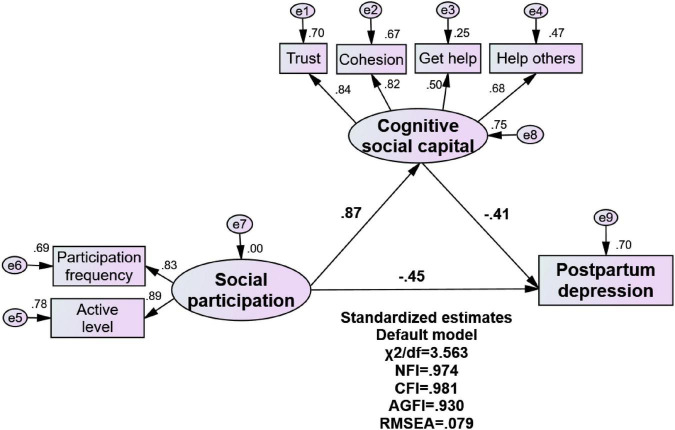
The model of the mediating role of cognitive social capital between social participation and postpartum depression symptoms.

Figure 2 depicts the mediation models that led to PPD symptoms and the standardized coefficients for all variables. Table 5 shows the results of the significant regression and correlation paths of SEM, which were significantly predicted by SP and CSC. The standardized direct effect value of CSC on PPD symptoms was -0.412 (*P* < 0.001), and the standardized direct effect value of SP on PPD symptoms was -0.453 (*P* < 0.001). SP significantly predicted CSC, and the standardized direct effect of SP on CSC was 0.865 (*P* < 0.001). According to the results of the bootstrap mediation effect test, the bias-corrected 95% CI of path 1 was 0.812–0.908, and the bias-corrected 95% CI of path 2 was -0.62 to -0.218. This confirmed that the indirect effect of SP on PPD symptoms through CSC was significant. The standardized indirect effect of SP on PPD symptoms through CSC was -0.356 (bias-corrected 95% CI -0.55 to -0.19). Further, the standardized total effect of SP on PPD symptoms was -0.809, with indirect effects accounting for 44.00% of the total effect.

**TABLE 5:** Standardized bootstrap mediation effect test.

Mediation effect path	Standardized estimate	SE	Bias-corrected 95% CI			Percentile 95% CI		
			Lower	Upper	*P*	Lower	Upper	*P*
Path 1	0.865	0.024	0.812	0.908	0.001	0.816	0.911	<0.001
Path 2	–0.412	0.103	–0.62	–0.218	0.001	–0.614	–0.215	0.001
Path 3	–0.453	0.103	–0.647	–0.24	<0.001	–0.65	–0.247	<0.001
Path 4	–0.356	0.092	–0.55	–0.19	0.001	–0.544	–0.185	0.001

Path 1: SP-CSC; Path 2: CSC-PPD; Path 3: SP-PPD; Path 4: SP-CSC-PPD.

## Discussion

### Postpartum depression symptoms of lactating women in minority areas of southwest china

In this study, the continuous EPDS score was used to evaluate PPD symptoms of lactating women in minority areas of southwest China. Our findings show that the mean PPD symptoms score was 7.87, and the prevalence of PPD symptoms was 16.46%, compared with 7.8 and 23.2% reported in other studies on lactating Chinese women based on the same standards (Huang et al., 2020; Liu et al., 2020; Zhang et al., 2021). The prevalence of postpartum depression in central Vietnam and Vientiane Laos, which are close to the Guangxi Zhuang Autonomous Region, is 18.1 and 31.8%, respectively (Murray et al., 2015; Inthaphatha et al., 2020). The overall prevalence of PPD symptoms reported in this study was closer to the estimated value (14.6%) of lactating women in northwestern minority areas. However, the prevalence of PPD symptoms in minority lactating women was 13.67%, which was slightly lower than the 16.1% prevalence in Kazak women in northwestern areas (Chen et al., 2018). Although some studies have shown that PPD symptoms of minority women were higher than those of Han women, our study did not show a significant difference in the ethnic distribution of PPD symptoms (Huang et al., 2020). One possible explanation is that ethnic integration eliminates the differences in the level of PPD symptoms among lactating women of different ethnic groups (Shakeel et al., 2018). As a multi-ethnic region, the Guangxi Zhuang Autonomous Region has implemented a policy of multi-ethnic integration for a long time. Minorities represented by the Zhuang live together with Han in the same geographical environment, and there is rarely ethnic segregation at present. Long-term ethnic integration has formed a common economy, reproductive culture and living habits with local characteristics, which may be the reason why there is no significant difference in PPD symptoms among lactating women of different ethnic groups.

### Demographic and lactating factors predicting postpartum depression symptoms

Hierarchical regression analysis was performed to confirm the correlations of sociodemographic characteristics, breastfeeding factors, and SC with PPD symptoms. The symptoms of depression gradually increased with age in lactating women, consistent with previous studies from Spain, Sweden, and Guangzhou China (García-Blanco et al., 2017; Silverman et al., 2017; Xiong and Deng, 2020a). One possible explanation is that elderly mothers’ family responsibilities have increased, and they face additional burdens to contribute to family finances. Additionally, elderly mothers face greater risks of childbirth, anxiety about postpartum rehabilitation and body management, and anxiety about the baby’s health. Thus, compared to young mothers, they may be under greater psychological pressure (García-Blanco et al., 2017; Strelow et al., 2018; Xiong and Deng, 2020a). Further, we found a strong negative association between the medical insurance of lactating women (regardless of type) and PPD symptoms, compared to people without medical insurance. Similarly, previous studies have shown that a lack of medical insurance or medical assistance plans prevents postpartum mothers from receiving treatment and mental health support, which would increase the possibility of PPD (Bhat et al., 2020; Epperson et al., 2020; Laudi and Peeples, 2020).

Among the factors during the breastfeeding period, the duration of breastfeeding, primary caregiver, and length of maternity leave were important predictors of PPD symptoms. Our research results showed that with an increase in breastfeeding time, the severity of PPD symptoms increased if lactating women did not receive care support from other family members but only takes care of the baby by themselves, consistent with a previous study (Dol et al., 2021; Peng et al., 2021). However, it is worth noting that longer maternity leave for lactating women helps relieve the pressure of postpartum depression, similar to the results of studies in the United States (Dagher et al., 2014; Kornfeind and Sipsma, 2018). Combining this point, it is not difficult to understand that, compared with regular occupations, freelance plays a protective role in PPD symptoms. China’s labor law regulations stipulate that employers give approximately 98 days of maternity leave to lactating women for physical recovery and breastfeeding infants. However, once the maternity leave is over, lactating women will face the double pressure of taking care of the baby and returning to work (Chong et al., 2016; Basrowi et al., 2018). In the Chinese culture, there is a long period of breastfeeding habits (up to 1 year or more). However, if they lack support from family members, lactating women have to frequently travel to and from home and the workplace, which consumes their time and energy, and creates great psychological pressure on them.

### Social capital factors predicting postpartum depression symptoms

After adjusting for the control variables (demographic and social characteristics and breastfeeding factors), SP, SR, and ST were negatively correlated with PPD symptoms scores, and the explained variance increased to 71.6% in Model 3. This indicated that SC was associated with PPD symptoms of lactating women in minority areas of Southwest China. Our research found that the impact of SSC is relatively vague; in which SP had a significant impact on PPD symptoms, but SN did not. Our findings are supported by other studies (Mann et al., 2008; Keefe et al., 2016; Zhou et al., 2017b), but also differ from the findings of some research, such as a study conducted in Ghana showing that active SP could increase stress among participants, leading to depression and other common mental health disorders. This contradictory result may suggest a unique interpretation that social networks as the static structures of SC especially for breastfeeding women from ethnic minorities in southwest China. Only through the frequency of SP and active roles can the dynamic interactions of mothers’ social interactions be formed, thereby exerting the utility of SSC. Participating in community activities can provide potential continuing benefits for lactating women. Therefore, maternity hospitals in China could organize relevant organizations for guidance on feeding norms, such as maternal and child health centers, helping them disperse the psychological pressure of long-term care of the baby (Strange et al., 2016; Levin, 2021; Dauda and John-Akinola, 2022).

Our results showed that CSC plays a protective role in postpartum depression; similar findings were reported in previous studies by Kritsotakis, Zhou, and Murray, the higher the level of CSC, the lower level of depression symptoms (Kritsotakis et al., 2013; Murray et al., 2015; Zhou et al., 2018). One possible reason is that there still exists a community environment linked by blood ties, such as clans and families in ethnic minority areas in China. Because of the long-term ethnic integration policy, multi-ethnic mixed communities show ethnic diversity and integration, and the cultural foundation of trust and reciprocity (Shakeel et al., 2018). In communities with higher levels of ST and SR, mothers can more conveniently communicate health knowledge about parenting and emotional counseling with other mothers to reduce their psychological stress. ST increases the exchange of emotions among individuals, relatives, and neighbors (Amegbor et al., 2020). SR means that people can feel the goodwill and help in their surrounding environment, and “Being of value to others” boosted people’s belief in their strength (Smit et al., 2021).

Furthermore, we found that SR was more important than ST for PPD symptoms among lactating women in minority areas, consistent with other research results on antenatal depression among Chinese primiparas (Zhou et al., 2017b). It might be that frequent population migration in China’s social transformation is causing a crisis of trust in people’s interpersonal relationships, reducing people’s general sense of trust (Yao et al., 2017). Another possible reason is that reciprocity may be a more important antecedent than trust propensity in social interactions (Aggarwal et al., 2016). As explicit behaviors, reciprocity actions, such as gift-giving and mutual aid, are easier to satisfy than trust (Mathews, 2017). SR makes it easier for lactating women in minority areas to feel happy by receiving social support from people around them. In the typical phenomenon of traditional Chinese culture, relatives, friends, and neighbors will bring gifts and blessings for the newborn and their mothers during special events such as the birth of a baby, the full moon, and the hundred days of the baby, which is also common in minority areas in southwest China (Li, 2020; Wang, 2021).

An interesting finding was that SEM showed a mediating effect of CSC on the relationship between SP and PPD symptoms. The mediation effect rate was 44.00%, confirming that SP indirectly affected PPD symptoms through CSC. This may be attributed to the gradual development of China’s community service system and the increasing sense of community belonging among residents (Lu and Peng, 2019). A study in Kenya showed that mothers who often participate in community activities to improve their ability to care for children have lower levels of depression. This connection is mediated by increased cohesion and trust (Goodman et al., 2020). Some scholars have suggested that regular attendance and group practices may enhance entitativity (group cohesion) by regularly expressing shared values, interdependence, and more regular identities; the higher the cohesion, the higher people’s expectations that their needs may be met, and the higher the likelihood of expressing trust (Hogg and Reid, 2006; Goodman et al., 2020). Participation can satisfy members’ attachment needs, including connection, emotional attachment, and acceptance, allowing participants to spread risks and share functional, emotional, and psychological loads with their peers (Beckes and Coan, 2011; Goodman et al., 2020).

## Limitations

First, this study was not based on clinical diagnosis to assess PPD, but rather on a self-report scale (EPDS); a continuous score was used to measure levels of PPD symptomatology. However, it cannot be used to confirm PPD in women clinically. Second, the study only conducted a cross-sectional survey design, which could not test the causal relationship between SC and PPD symptoms; therefore, future prospective studies are needed. Finally, the sample was from the Guangxi Zhuang Autonomous Region in southwest China, which may limit the generalizability of the findings. Thus, further research is needed to confirm whether the current research results apply to other regions.

## Conclusion

To the best of our knowledge, this study is the first to investigate the relationship between SC and PPD symptoms among lactating women in ethnic minority areas in China. Our study showed that elderly lactating women who are primary caregivers with no medical insurance, fixed occupations, and short maternity leave were more likely to experience postpartum depression. Further, higher SC, including SP, ST, and SR was strongly associated with lower PPD symptoms, and CSC composed of ST and SR mediated the relationship between SP and PPD symptoms. These findings provide new evidence for the social capital theory applied to the field of postpartum mental health. It also helps policymakers to formulate preventive interventions to enhance SSC especially SP and CSC, to effectively alleviate PPD symptoms in lactating women. Moreover, favorable conditions should be provided to promote the SP of lactating women. These include extending maternity leave, providing family support, and creating peer support groups. Finally, it is important to cultivate lactating women’s SC via community centers and hospitals. Healthcare professionals and community workers should regularly conduct activities to promote mental health awareness and provide information about postpartum health and baby care. Moreover, timely social support through psychological counseling should also be provided.

## Data availability statement

The datasets used and analyzed during the current study are available from the corresponding authors on reasonable request.

## Ethics statement

The study meets the ethical guidelines of the Helsinki Declaration and it was approved by the Medical Ethics Committee of Guilin Medical University. Written informed consent was obtained from participants before participation in this study.

## Author contributions

QHW and WXS were responsible for the overall design of the research. YHQ analyzed the results and drafted the manuscript. PFG and SCJ substantially contributed to data acquisition. JCL, XZ, and RZW assisted with the literature review. JHW and HL contributed to the interpretation of the results and the writing of the manuscript. FY, JJL, and KXW revised the manuscript. All authors contributed to this manuscript and approved the current version for publication.

## Abbreviations

PPD: postpartum depression
EPDS: Edinburgh Postnatal Depression Scale
SC: social capital
SSC: structure social capital
CSC: cognitive social capital
ST: social trust
SR: social reciprocity
SP: social participation
SN: social network.
